# Dose-dependent impact of statin therapy intensity on circulating progenitor cells in patients undergoing percutaneous coronary intervention for the treatment of acute versus chronic coronary syndrome

**DOI:** 10.1371/journal.pone.0267433

**Published:** 2022-05-19

**Authors:** Roberta Florescu, Elisa Liehn, Nicole Schaaps, Jörg Schröder, Mohammad Almalla, Sebastian Mause, Anne Cornelissen, Felix Vogt

**Affiliations:** 1 Department of Cardiology, University Hospital Aachen, RWTH Aachen University, Aachen, Germany; 2 Institute for Molecular Medicine, University of South Denmark, Odense, Denmark; 3 National Institute of Pathology “Victor Babes”, Bucharest, Romania; University of Campania Luigi Vanvitelli Department of Translational Medicine: Universita degli Studi della Campania Luigi Vanvitelli Dipartimento di Scienze Mediche Traslazionali, ITALY

## Abstract

**Background:**

By low-density lipoprotein (LDL) reduction, statins play an important role in cardiovascular risk modification. Incompletely understood pleiotropic statin effects include vasoprotection that might originate from mobilisation and differentiation of vascular progenitor cells. Data on the potentially differential impact of statin treatment intensity on circulating progenitor cells in patients undergoing percutaneous coronary intervention (PCI) are scarce. This study examines the potential association of different permanent statin treatment regimens on circulating progenitor cells in patients with coronary syndrome.

**Methods and results:**

In a monocentric prospective all-comers study, 105 consecutive cases scheduled for coronary angiography due to either (A) non-invasive proof of ischemia and chronic coronary syndrome (CCS) or (B) troponin-positive acute coronary syndrome (ACS) were included. According to the 2018 American College of Cardiology Guidelines on Blood Cholesterol, patients were clustered depending on their respective permanent statin treatment regimen in either a high- to moderate-intensity statin treatment (HIST) or a low-intensity statin treatment (LIST) group. Baseline characteristics including LDL levels were comparable. From blood drawn at the time of PCI, peripheral blood mononuclear cells were isolated, cultivated and counted and, by density gradient centrifugation, levels of circulating progenitor cells were determined using fluorescence-activated cell sorting (FACS) analysis. In ACS patients both absolute and relative numbers of circulating early-outgrowth endothelial progenitor cells (EPCs) concurrently were significantly lower in the HIST group as compared to the LIST group. This effect was more pronounced in ACS patients than in CCS patients. Both in ACS and CCS patients, HIST caused a significant reduction of the number of circulating SMPCs.

**Conclusions:**

In patients undergoing PCI, a dose intensity-dependent and LDL level-independent pro-differentiating vasoprotective pleiotropic capacity of statins for EPC and SMPC is demonstrated.

## Introduction

Statin treatment plays a pivotal role in the prevention of coronary artery disease (CAD) both in patients with acute coronary syndrome (ACS) as well as chronic coronary syndrome (CCS). Endothelial dysfunction plays a crucial role in the initiation and progression of CAD and is potentially modulated by changes in number and function of circulating progenitor cell populations released from the bone marrow. Derived from the bone-marrow, circulating early-outgrowth endothelial progenitor cells (EPCs) substantially contribute to vascular repair [1–3]. Due to their ability to proliferate, migrate to the site of vascular injury, and differentiate into mature vascular endothelium EPCs facilitate re-endothelialisation [1–3]. Numerous studies focus on the phenotypic and functional properties of EPCs and the role of EPCs in clinical conditions such as CAD or myocardial infarction (MI) [4] attracts substantial attention. Thus, a growing body of evidence suggests that cardiovascular risk factors and risk factor-modulating drug therapies affect the number and properties of EPCs [3, 5]. However, data on the potential LDL level-independent impact of statin treatment intensity on EPCs in patients undergoing percutaneous coronary intervention (PCI) are scarce.

Since the discovery of circulating EPCs, research has been aimed at the characterisation of the various subtypes of cells that differentiate from circulating progenitors, the development of protocols for EPC characterization and quantification, and the clarification of the clinical relevance of EPCs. With the expansion of the research field, the related nomenclature has become increasingly complex and the optimal definition of EPCs still causes ambiguity. Generally, two methods are used to identify EPCs. One of the approaches is based upon the confirmation, after a defined period under angiogenic culture conditions, of the specific mature endothelial morphology of endothelial precursors [6]. Based on time of occurrence in culture on fibronectin coating, a differentiation can be made between: (A) endothelial cell colony-forming units (EC-CFU), also referred to as both EPC-CFU and early outgrowth endothelial cells (EOCs) with elongated spindle shape morphology on day 5 until 7 and (B) endothelial colony-forming cells (ECFCs) of late outgrowth colonies that occur at 2–3 weeks in culture. Another widely employed method is to specifically identify cells displaying surface markers that indicate an endothelial phenotype using fluorescence-activated cell sorting (FACS) [6]. Initially, EPCs were defined by expressing CD34 and kinase domain receptor (KDR) [7]. An additional marker, CD133, has been suggested for further identification of EPC [8, 9]; however, several authors found that CD133 identifies a purely haematopoietic cellular line and that these cells are in fact not capable of forming a true endothelial phenotype [10, 11]. Thus, in general, EPC are characterised by expression of CD34 and KDR, as well as by their ability to take up acetylated LDL (Ac-LDL) and to bind an endothelial specific lectin [9, 12, 13].

Due to the prevailing hypothesis that smooth muscle progenitor cell (SMPC) recruitment may contribute to plaque stabilisation [14–16], SMPCs represent another intriguing subpopulation of circulating progenitor cells. In brief, three different methods have been described to identify SMPCs: (A) functional identification considering the specific ability to contract under stimulation with smooth muscle-specific pharmacological agonists, (B) use of in vitro culture conditions with collagen or fibronectin plate coating maintained in medium supplemented with platelet derived growth factor BB (PDGF-BB) [16–18] for morphological identification, and (C) FACS detection of distinct surface or intracellular marker expression [16–18]. While several studies have aimed to elucidate the potential contribution of SMPCs in the development of atherosclerotic lesions, data are scarce both on potential pleiotropic effects of statins on this cell population as well as on a potential general role of SMPCs in patients with CAD undergoing PCI.

This study aims to detect a potential impact of high- to moderate-intensity statin therapy (HIST) or low-intensity statin therapy (LIST) treatment regimens on EPCs and SMPCs in patients after PCI for the treatment of ACS or CCS, respectively. Thus, we hypothesize that intensified statin treatment has a capacity to optimize endothelial function and plaque morphology by modifying circulating progenitor cell migration and maturation, thereby potentially improving clinical outcome of CAD patients.

## Methods

### Study population and design

In a monocentric prospective all-comers study design, consecutive cases with an indication for coronary angiography due to either ACS with positive blood marker troponin or CCS with non-invasive proof of ischemia were included. Prior to inclusion, informed consent was obtained from all patients. The study was approved by the Ethics Committee of the Medical Faculty at University Hospital Aachen (EK 164/07). All patients that received at least one coronary stent were included. All patients were on permanent statin treatment due to known CCS, hyperlipidaemia, or documented peripheral artery disease beginning at least four weeks before inclusion. According to the statin efficacy as defined in the 2018 American College of Cardiology Guidelines on Blood Cholesterol [19], patients were clustered in a HIST or LIST therapy group, respectively. Immediately after PCI, blood samples were collected under sterile conditions through a central venous sheath or from a large lumen peripheral venous line for further processing.

### Selection and culture of attached EPCs and SMPCs

Twenty mL whole blood samples were taken from each patient directly after PCI using natriumcitrate-dextrane as anticoagulant and immediately processed. EPCs and SMPCs were cultured as previously described [20, 21]. In short, peripheral blood mononuclear cells (PBMCs) were separated using density gradient centrifugation and counted. To select and cultivate EPCs, 10^6^ PBMCs were plated in 6-well plates coated with 200μg/mL fibronectin (Tebu-Bio, Cat-No 2003, Norwood, MA) and maintained in Endothelial Cell Growth Medium MV2 Kit (PromoCell, Heidelberg, Germany). For SMPC selection and cultivation, 10^6^ PBMCs were plated in 6-well plates coated with 80μg/mL Collagen G (Biochrom AG, Berlin, Germany) and maintained in Dulbecco´s Modified Eagle Medium (NUT MIX F12—Invitrogen, Auckland, TX) supplemented with 10% fetal calf serum (FCS) (Invitrogen, Oregon) and 10ng/mL PDGF-BB (Provitro, Berlin, Germany). On day 4 the non-adherent cells were removed; EPC medium was replaced completely while half of the SMPC medium was changed. At day 7 in culture, PBMCs developed the EPC-specific elongated spindle shape morphology or the SMPC “hill and valley morphology”, respectively. Thus, the fraction of EPCs and SMPCs, respectively, derived from a defined PBMC amount was measured by counting the adherent cells. In addition, the absolute whole blood EPC concentration was calculated depending on the number of PBMCs per ml in the respective blood volume.

### Identification and quantification of EPCs and SMPCs

EPCs were quantified by FACS analysis as previously described [20, 21]. Briefly, sodium citrate anticoagulated peripheral blood together with antibodies (100μL blood/double staining) was incubated for 30 minutes. For detection of EPCs, fluorescein isothiocyanate anti human CD34 (FITC-labelled, BD Pharmingen^TM^, California) and phycoerythrin anti human KDR (PE-labelled, BD Pharmingen^TM^) antibodies were used. For detection of SMPCs, antibodies against human CD34 (FITC-conjugated, BD Pharmingen^TM^) and PDGF receptor (PE labelled, BD Pharmingen^TM^) were used. FITC- or PE-conjugated isotype control antibodies (Iso FITC mouse IgG1K, Iso mouse IgG2aK PE, mouse IgG1 Isotype PE) from the same manufacturer served as controls. Each analysis included at least 10.000 events within the lymphocyte gate. Data were processed using Cellquest software (Version 5.1, BD Biosciences, USA).

For further phenotype confirmation, cultivated EPCs were fixed using 2% paraformaldehyde for 10 minutes and stained with FITC-labelled lectin Ulex europaeus agglutinin I (10mg/mL; Sigma, NY) for 1h. Cells were incubated with 1,19–dioctadecyl–3,3,39,39–tetramethyl indocarbocyanine–labelled acetylated low-density lipoprotein (Dil-Ac-LDL) (2.4mg/mL; *Biochrom* AG) at 37°C for 2h to detect the uptake of Dil-Ac-LDL. Samples were viewed with an inverted fluorescent microscope (Leica). Dual-stainined cells positive for both lectin and Dil-Ac-LDL were judged as EPCs.

### EPC adhesion assay

At day 7 in culture, 20.000 cells/well were seeded on 96-well plates coated with 200μg/mL fibronectin (TebuBio) in Endothelial Cell Growth Medium MV2 Kit (PromoCell) and incubated at 37°C. After 30 minutes incubation time, the supernatant was carefully discarded and the wells were washed 3 times with phosphate buffered saline (PBS) (Biochrom AG). EPC adhesion was quantified using the fluorescence based CyQuant Assay Kit (Invitrogen) according to the manufacturer’s instructions. In addition, data was normalised on whole blood EPC concentration.

### Statistical analysis

Data are expressed as mean ± standard error of the mean (SEM). Comparisons between groups were analysed by *t*-test (two-sided) and ANOVA for multiple comparisons with normally distributed variables followed by Fischer’s LSD test. A point biserial correlation was used to analyse the association between progenitor cells and cardiovascular risk factors. A multivariate analysis of variance was performed to evaluate the risk factors for CAD. P values less than 0.05 were considered as statistically significant. All statistical analyses were performed with Prism (Version 8.4.1, GraphPad Software, San Diego, CA).

## Results

105 consecutive cases (82 males) were included. All patients have been under statin therapy for at least four weeks prior to PCI. Baseline characteristics including LDL levels were comparable (Table 1).

**Table 1:** Baseline clinical characteristics of patients.

	All patients	HIST ACS	HIST CCS	LIST ACS	LIST CCS
**Age (years)**	63.4±1.1	60.5±2.2	63.7±2.0	66.3±2.1	63.7±2.3
**Men, n (%)**	82 (78.1)	19 (79.1)	27 (79.4)	13 (68.4)	23 (82.0)
**Medical history, n (%):**					
**Hypertension**	80 (76.2)	19 (79.1)	27 (79.4)	14 (73.6)	20 (71.4)
**Diabetes mellitus**	30 (28.6)	7 (29.1)	8 (23.5)	6 (31.5)	9 (32.1)
**Current smoking**	45 (42.9)	14 (58.3)	13 (38.2)	5 (26.3)	13 (46.4)
**Family history of CAD**	36 (34.3)	7 (29.1)	17 (50.0)	7 (36.8)	14 (50.0)
**Body mass index (kg/m^2^)**	28.2±5.2	30.7±1.6	27.7±0.6	26.0±0.9	28.5±0.8
**LDL-Cholesterol (mg/dL)**	116.6±3.9	119.2±8.2	112.3±6.0	126.1±13.4	112.7±5.5

Data are expressed as mean±SE or n (%).

No correlation was found between EPC or SMPC count, respectively, and cardiovascular risk factors (r from -0.21 to 0.19) (S1 Fig). 58 patients were on HIST and 47 patients on LIST (Table 2).

**Table 2:** Patient allocation to different statin regimen.

Statin intensity	Type and dosage of statin	Number of patients (%)
	Atorvastatin 20mg	3 (5.4)
	Atorvastatin 40mg	13 (23.6)
**HIST**	Fluvastatin 80mg	3 (5.4)
	Simvastatin 20mg	12 (21.8)
	Simvastatin 40mg	26 (47.2)
	Simvastatin 80mg	1 (1.8)
	Fluvastatin 20mg	2 (4.2)
**LIST**	Fluvastatin 40mg	9 (17.0)
	Pravastatin 20mg	1 (2.1)
	Simvastatin 10mg	35 (74.4)

HIST (high- to moderate-intensity statin treatment) or LIST (low-intensity statin treatment).

There was no significant difference between males and females regarding EPC counts (10360±1574 vs. 11710±2127, p = 0.67) and SMPC counts (43790±4453 vs. 31570±7389, p = 0.19) (S2 Fig).

**Fig 1:**
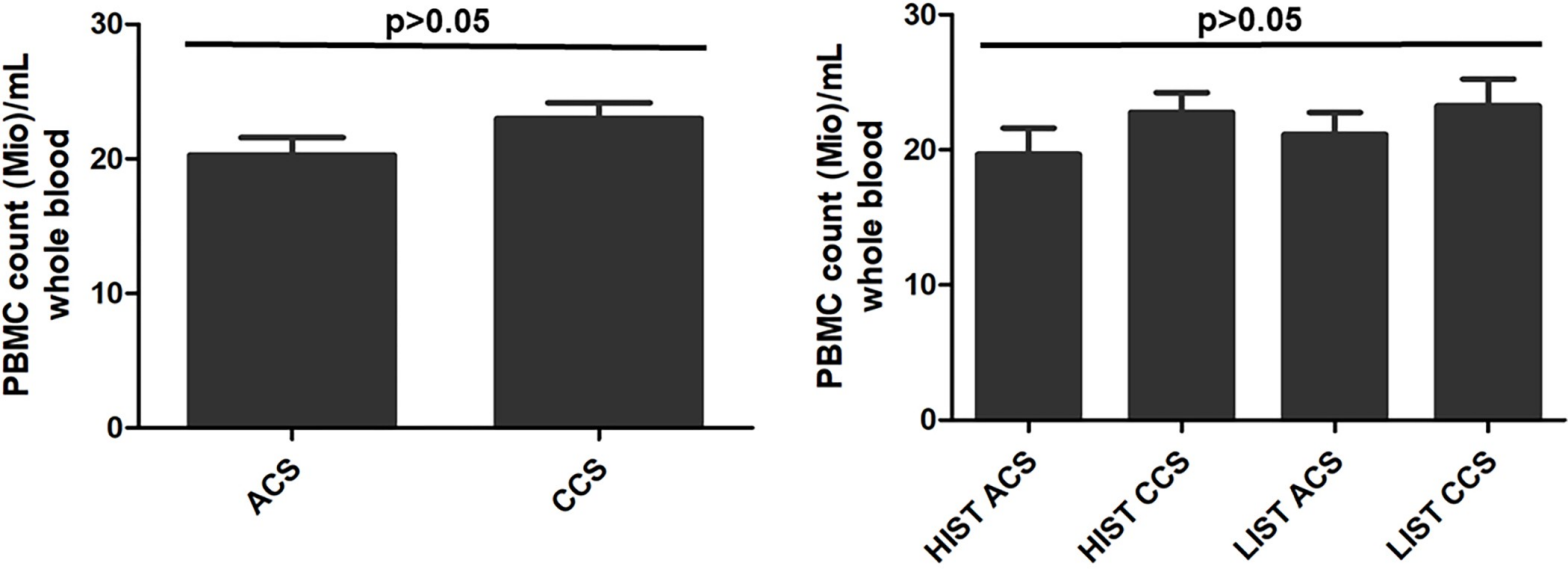
Similar whole blood PBMC counts. PBMC counts were similar in all ACS versus CCS patients (left); also, PBMC counts were similar in ACS versus CCS patients stratified by permanent statin treatment intensity (HIST: high- to moderate-intensity statin treatment; LIST: low-intensity statin treatment) (right).

Under HIST versus LIST and ACS versus CCS (Fig 1), respectively, there was no significant difference in absolute whole blood PBMC concentration.

**Fig 2:**
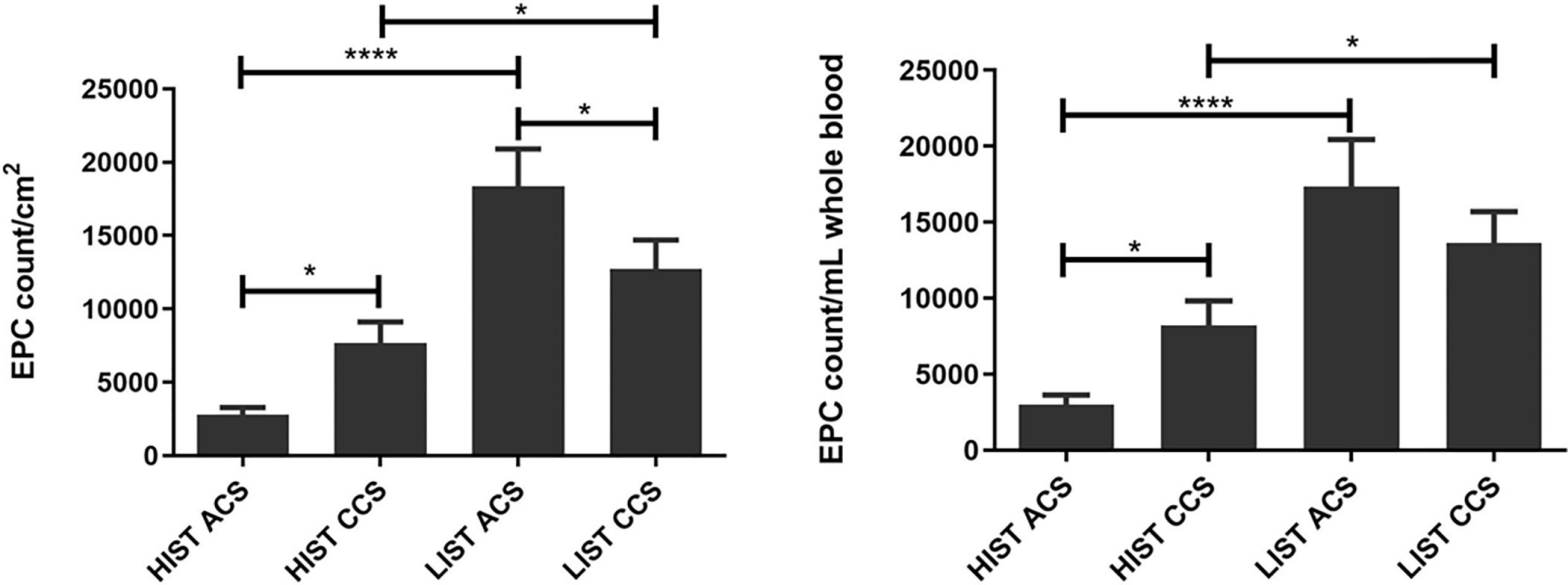
Reduced relative and absolute EPC counts under HIST. EPC counts per cm^2^ (relative numbers) (left) and per mL whole blood (absolute numbers) (right) in each group depending on the clinical status and statin potency.

In the clinical context of ACS, patients receiving HIST compared to LIST showed significantly decreased counts of absolute (3122±648 vs. 16394±3080, p<0.0001) and relative EPC numbers (2803±476 vs. 18365±2532, p = 0.0230). Similarly, in the clinical context of CCS, patients receiving HIST revealed significantly lower EPC counts compared to patients receiving LIST both in relative (7676±1428 vs. 12748±1950, p = 0.0216) and absolute numbers (7958±1599 vs. 13645±2062, p = 0.0230) (Fig 2).

**Fig 3:**
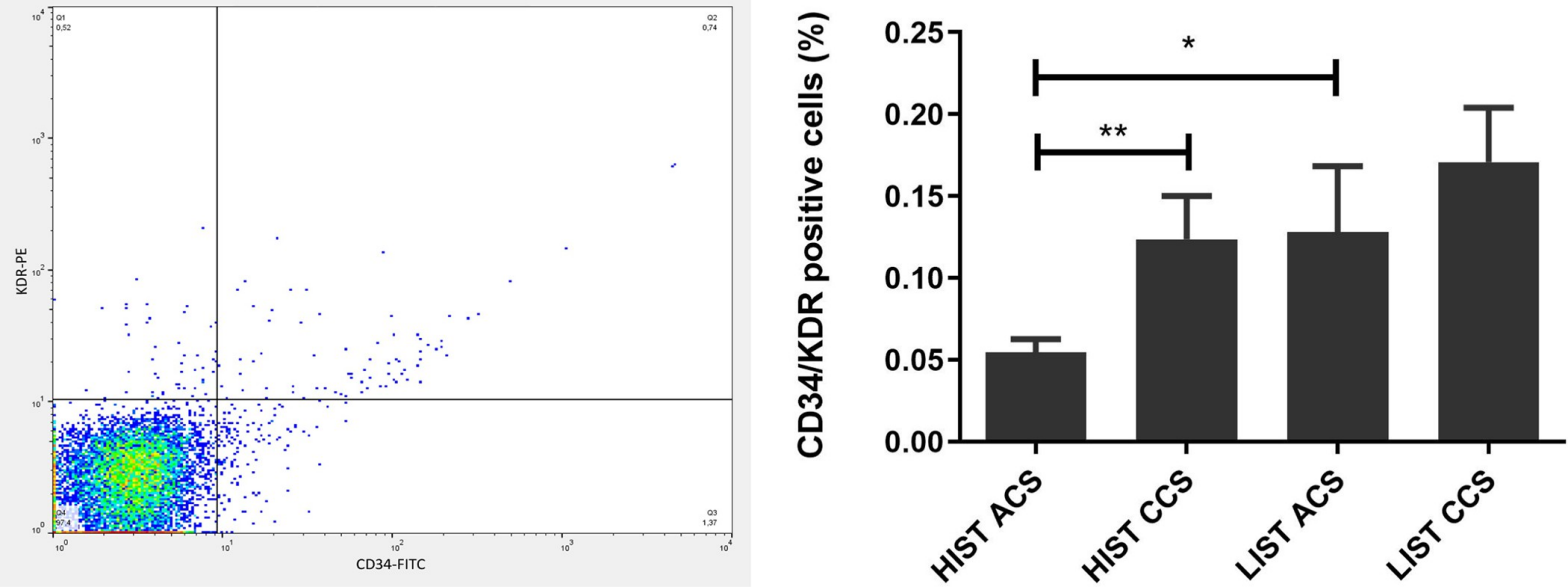
Reduced percentage of CD34+/KDR+ cells under HIST. FACS analysis with double fluorescence with PE-labelled KDR and FITC-labelled CD34 antibodies. Double positive cells are indicated in the upper right quadrant (left).

In patients with ACS, FACS analysis revealed lower percentages of CD34^+^ KDR^+^ EPC under HIST compared to LIST (0.055±0.008 vs. 0.137±0.037, p = 0.0097). In cases treated with HIST the percentage of CD34^+^ KDR^+^ EPC was lower in ACS compared to CCS (0.0550±0.008 vs. 0.1011±0.010, p = 0.0012) (Fig 3).

**Fig 4:**
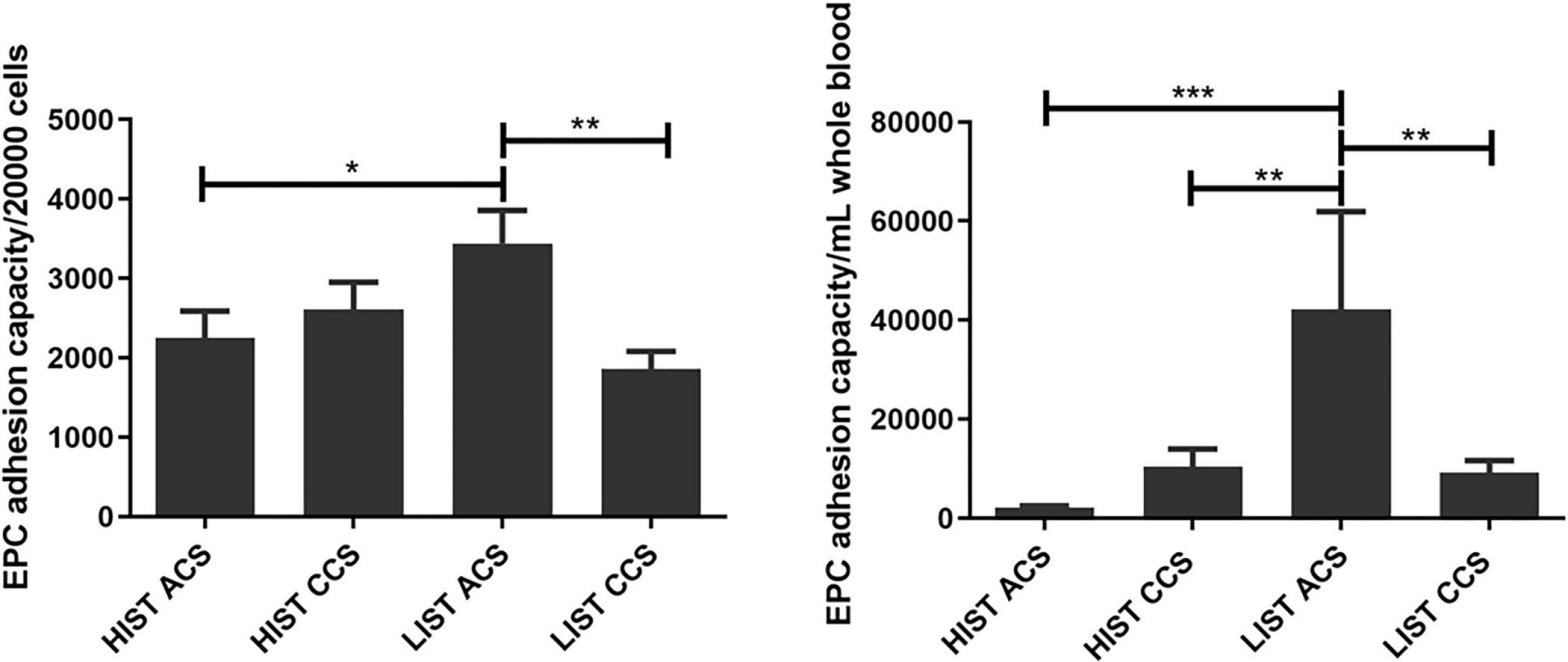
Pronounced EPC adhesion capacity in ACS patients under LIST. EPC adhesion capacity/20000 cells (fluorescence intensity at 485 and 535nm) (left) and normalised EPC adhesion capacity/mL whole blood (right) reveal markedly higher adhesion capacity in LIST ACS patients.

In ACS patients, EPC adhesion capacity was more pronounced under LIST compared than HIST (42180±19710 vs. 2086±446, p = 0.0157). Moreover, under HIST, a weaker capacity to adhere was observed in ACS compared to CCS (2086±446 vs. 10380±3583, p = 0.0293), while under LIST, adhesion capacity was markedly higher in ACS compared to CCS patients (42180±19710 vs. 9158±2493, p = 0.0394) (Fig 4).

**Fig 5:**
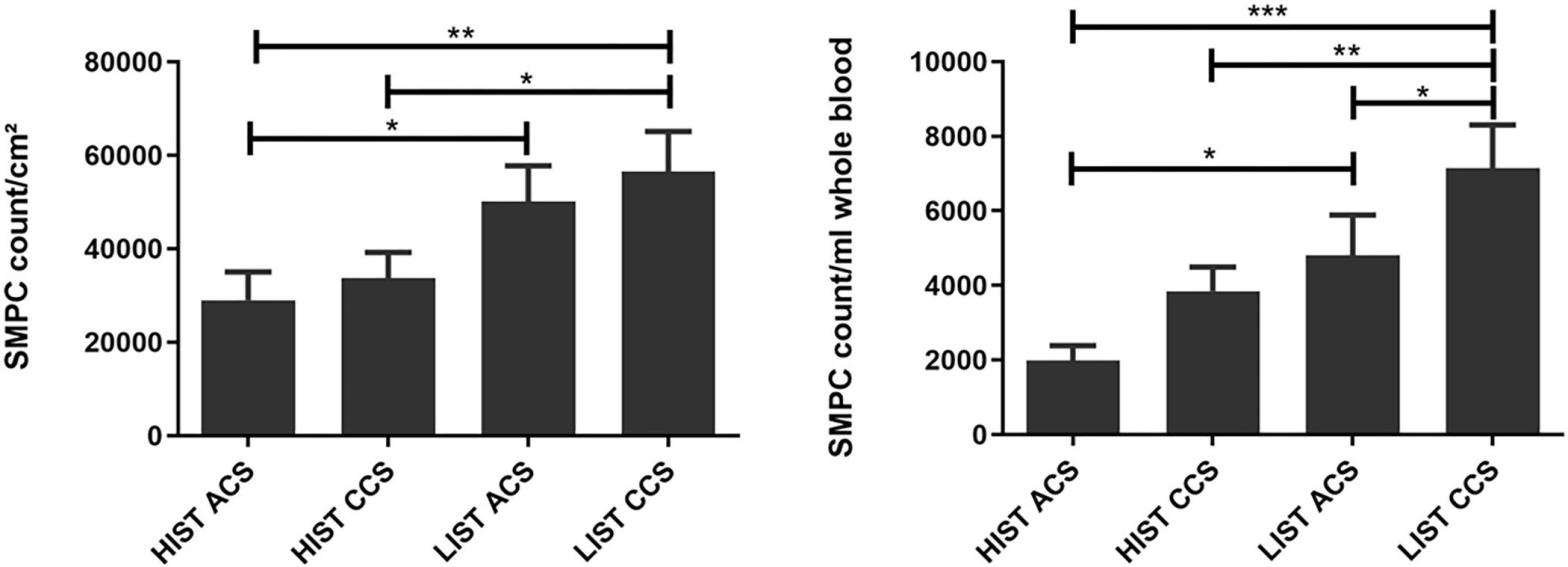
Suppressed SMPC counts in patients under HIST. Relative (left) and absolute (right) SMPC counts revealed significantly lower SMPC levels in patients treated with HIST.

Both ACS and CCS patients exhibited decreased numbers of absolute (ACS: 2262±472 vs. 4528±1050, p = 0.0400; CCS: 3846±648 vs. 7146±1160, p = 0.0102) and relative (ACS: 28970±6083 vs. 50100±7694, p = 0.0358; CCS: 33726±5481 vs. 56517±8584, p = 0.0184) SMPC counts under HIST compared to LIST (Fig 5).

**Fig 6:**
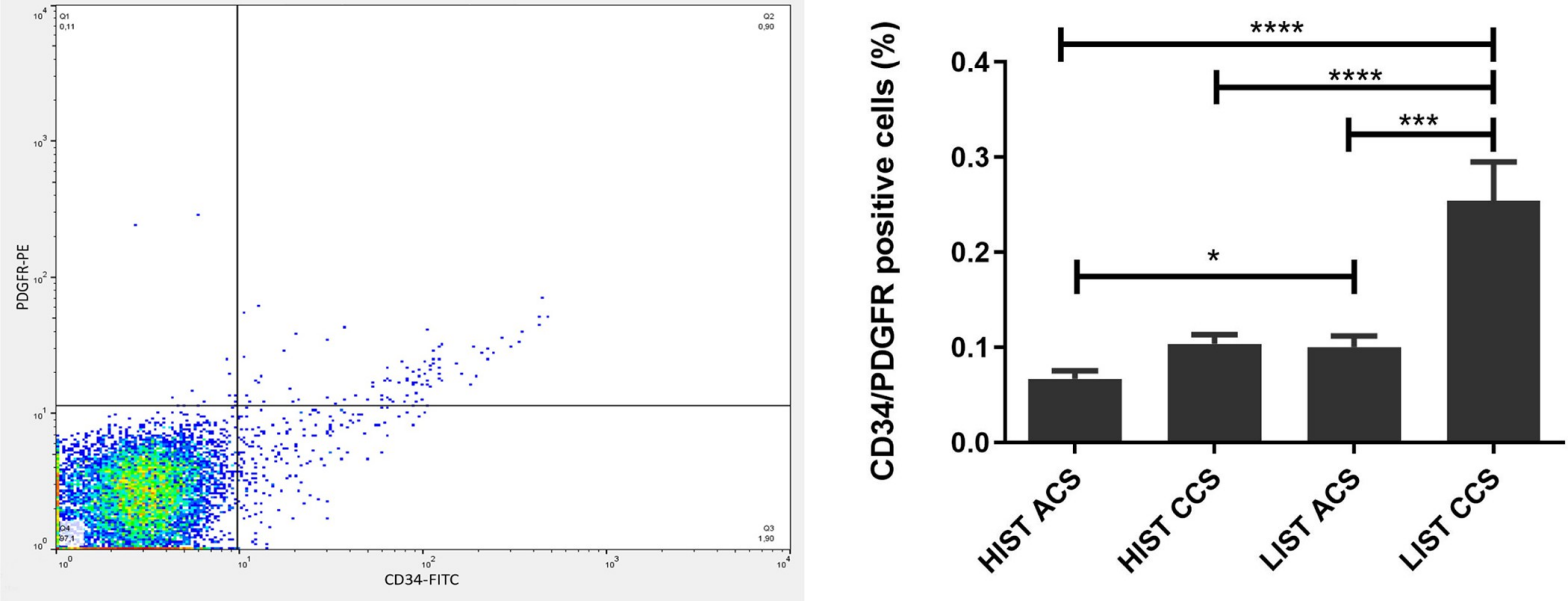
Depleted percentages of CD34+/PDGFR+ cells under HIST. Statin intensity impact on CD34^+^/PDGFR^+^ cells detected by FACS demonstrated lower percentages in patients under HIST (right). Double fluorescence with PE-labelled PDGFR and FITC-labelled CD34 antibodies; double positive cells are indicated in the upper right quadrant (left).

Similarly, irrespective of the clinical context, detection of SMPC surface markers revealed lower percentages of CD34/PDGFR positive cells under HIST compared to LIST (ACS: 0.03318±0.014 vs. 0.004638±0.061, p = 0.0241; CCS: 0.1043±0.009 vs. 0.2543±0.040, p = 0.0006) (Fig 6).

## Discussion

In patients undergoing PCI for ACS versus CCS treatment, the present study revealed an inverse correlation between statin treatment intensity and circulating EPC counts. In patients treated with HIST levels of circulating EPCs were significantly reduced; this effect was more pronounced in ACS than CCS patients. In the context of ACS, although the higher counts of EPCs in patients with LIST might be explained in part by an increase in the number of released PBMCs, EPC counts remained significantly higher compared to HIST after adjusting for mononuclear cells which might be explained by a HIST-facilitated maturation, migration and incorporation at sites of injury in the context of PCI. Moreover, both in ACS and CCS patients, HIST caused a significant reduction of the number of circulating SMPCs.

Statins are effectively used drugs in the treatment of CAD and several trials described pleiotropic effects including an anti-thrombotic effect and an improvement of endothelial function independent of the reduction of LDL-cholesterol [22–24]. In this study, the observed variations in EPC counts were independent from the lipid-lowering capacity of statins, as all groups had comparable LDL levels. In contrast to a number of available studies that have focused on short-term statin effects, the current study included only patients with long-term statin treatment and clustered them depending on the statin potency according to recent guidelines.

The functional capacity of bone marrow mononuclear cells was described to be significantly reduced in patients with CAD [4]. In patients with MI, EPC mobilisation to peripheral blood was first reported by Shintali et al. [25]. Myocardial ischemia was suggested to be the main stimulus for an increasing pool of circulating EPCs [26, 27] and, furthermore, an intracoronary rapid recruitment of EPCs in the early phase of MI was described as a response to reparative mechanisms [28].

Available data on pleiotropic effects of statins on EPCs are not restricted to mobilisation and homing to sites of vascular injury but include reports on statin-facilitated maturation and differentiation of EPCs [20, 21].

As the present study included exclusively patients with coronary syndrome undergoing PCI, iatrogenic vascular injury due stent induced endothelial denudation and endovascular laceration with subsequent vascular dysfunction is the major pathophysiological correlate in this population [6]. Bone marrow cells play an important functional role in myocardial infarction by releasing cytokines and growth factors and by reconditioning the population of progenitor cells. In selective areas of injured myocardium, the cells bear a homing capacity. Two important phases after PCI where previously described: the early response, within hours, and the late response, within days and months [6]. In the early response, endothelial cell colony-forming units (EC-CFU) and CD34+ cell populations are mobilised. An abnormal endothelial cell response might lead to delayed re-endothelialisation and restenosis. The late response within days to months is represented by the homing and proliferation of EPCs, leading to coverage of denuded surfaces. A potent response of the bone marrow might lead to a premature homing to the sites of vascular injury with subsequent early recruitment and integration of EPCs to the endothelium and, thus, promote restoration of normal endothelial function.

The studied population markedly differs in clinical characteristics and context from previous studies. Thus, during the pathway of EPC-facilitated improvement of coronary stent healing, the ability of EPCs to maturate, incorporate, and differentiate might be more crucial than their circulating numbers after mobilisation from the bone marrow in the studied population. Conclusively, we hypothesize that in the present PCI patient population, the high-potent statin dosages ultimately might improve the healing of stented coronary lesions by promoting maturation, incorporation, and differentiation of mobilized EPCs at coronary lesions that, in turn, might contribute to depletion of circulating EPC numbers.

Multiple studies have reported an association between cardiovascular risk factors and number of EPCs showing decreased counts in patients with diabetes, hypertension, hyperlipidaemia or smokers [3, 21, 29]. In addition, the mobilisation and the integration in injured endothelium but also the angiogenic capacity of the cells is affected. Lipid metabolism plays a decisive role in CAD, but also in the pathophysiology of EPCs. Once exposed to oxidized LDL, a dose-dependent impairment of the functional activity as well as an accelerated senescence of EPCs was observed and consequently could result in an up to 70% reduction in EPC counts [30, 31]. However, as the patients enrolled in this study showed no correlation between EPC counts and cardiovascular risk factors, the characteristics and counts of EPCs might be independent of this aspect.

By increased integrin expression, statins lead to an improvement of the migratory and adhesive activity of EPCs by upregulating the telomere-binding factor TRF2 in EPCs [32]. At the same time, via the PI 3-kinase/Akt pathway, EPCs differentiation was described to be another pleiotropic effect of statin therapy [33]. The adhesiveness of EPCs promotes the homing to sites of vascular injury. During PCI, balloon injury results in a rapid accumulation of fibronectin allowing the incorporation of EPCs at the altered vascular wall [34]. In this study, patients under HIST showed lower adhesiveness capacity of EPCs, potentially due to a premature maturation, differentiation and homing to the sites of vascular injury.

Although the mechanisms of statin therapy impact on SMPC are still unclear, our findings demonstrate a potentially beneficial pleiotropic effect of statin therapy in this specific patient subset. For the first time, SMPC levels were quantified on a larger cluster of patients with distinctive statin treatment and documented CAD. Both in ACS and CCS patients, HIST caused a significant reduction of the number of circulating SMPCs. Considering the fact that SMPC are involved in the progression of atherosclerosis after vascular injury [35, 36], circulating SMPC reduction due to HIST might reduce neointima hyperplasia and restenosis development and, thus, improve the clinical outcome of patients undergoing PCI.

Despite an increasing body of evidence on the efficacy of anti-inflammatory drugs for the treatment of CV disease, the current study did not include the analysis of a potential association between inflammatory markers and progenitor cells. Taking into consideration the substantial iatrogenic vascular injury during PCI, this aspect might be of crucial impact. As the present study aimed to determine the immediate changes after PCI associated with statin treatment, repeated sampling at later time points after PCI was not performed.

## Conclusions

In summary, our results reveal a dose intensity-dependent and LDL level-independent pro-differentiating vasoprotective pleiotropic capacity of statins for EPCs in patients undergoing PCI. The observed reduction of EPCs was more pronounced in ACS than in CCS patients and might be explained by a HIST-facilitated maturation, migration and incorporation at sites of injury in the context of PCI. The present findings concerning statin therapy interaction with SPMCs might point towards at potential pathway of HIST mediated restenosis suppression and endogenous regenerative statin effect after MI. These findings could potentially help to optimize drug treatment strategies in patients with CAD by distinct statin dosage and timing to improve endothelial function and plaque morphology and ultimately, clinical outcome.

## Supporting information

S1 Fig Analysis between cardiovascular risk factors, PBMC, EPC and SMPC. Pearson r correlation.
(PDF)

S2 Fig EPC/cm^2^ (left) and SMPC/cm^2^ (right) in males and females. No significant difference was observed between genders on EPC and SMPC counts.
(PDF)

S1 Table List of abbreviations. (DOCX)
