# Supplementary material for: Dose-dependent impact of statin therapy intensity on circulating progenitor cells in patients undergoing percutaneous coronary intervention for the treatment of acute versus chronic coronary syndrome
Source: PLoS One. 2022 May 19;17(5):e0267433. doi: 10.1371/journal.pone.0267433 (PMC9119492; doi:10.1371/journal.pone.0267433)
Supplement: S1 Fig — Pearson r correlation. (PDF) [file pone.0267433.s001.pdf]

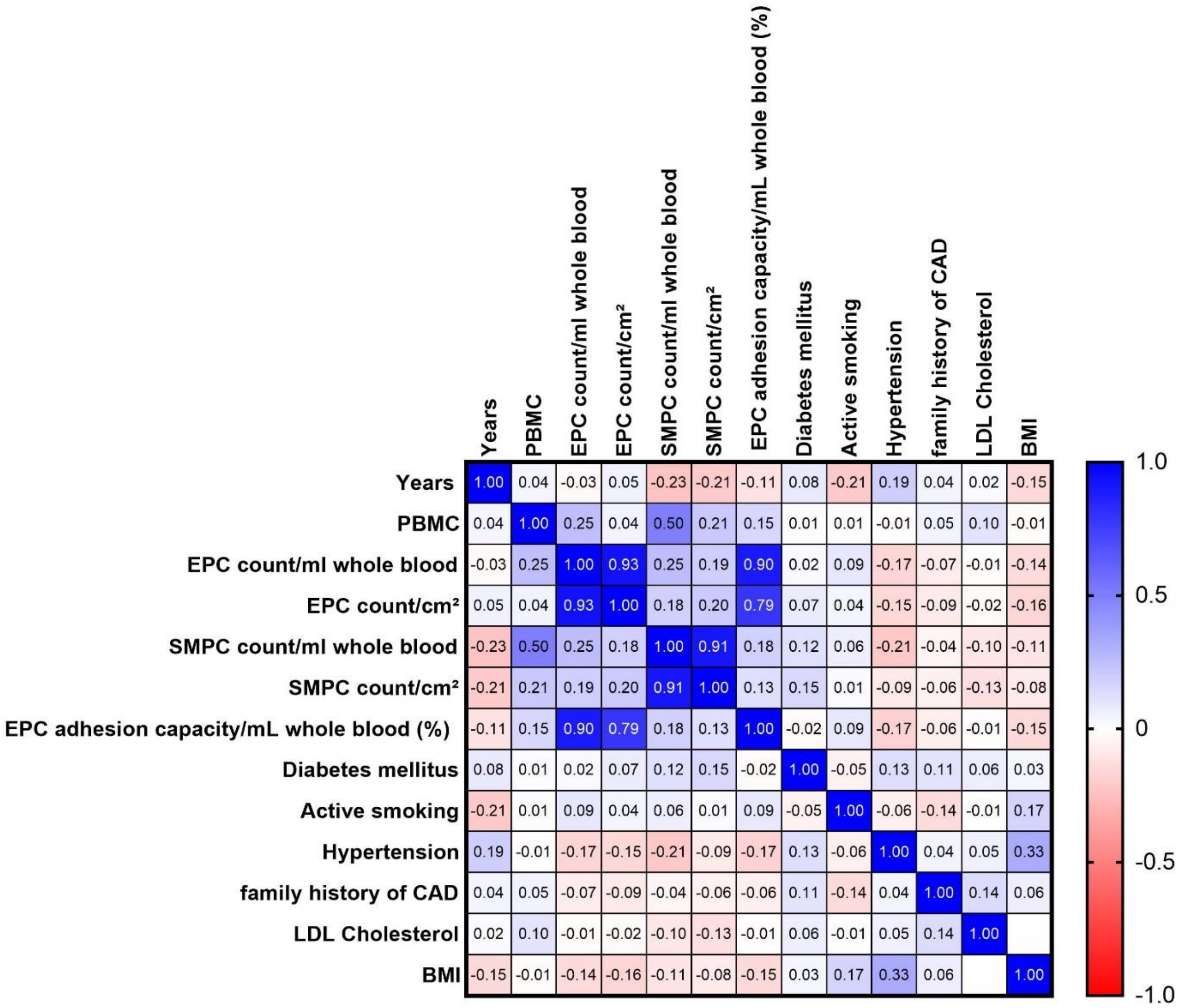

Figure S1. Analysis between cardiovascular risk factors, PBMC, EPC and SMPC. Pearson r correlation.
