# Supplementary material for: Dose-dependent impact of statin therapy intensity on circulating progenitor cells in patients undergoing percutaneous coronary intervention for the treatment of acute versus chronic coronary syndrome
Source: PLoS One. 2022 May 19;17(5):e0267433. doi: 10.1371/journal.pone.0267433 (PMC9119492; doi:10.1371/journal.pone.0267433)
Supplement: S1 Table — (DOCX) [file pone.0267433.s003.docx]

**Table S1. List of abbreviations**

|  |  |
| --- | --- |
|  |  |
| **Abbreviation** | **Complete Name** |
| LDL | low-density lipoprotein |
| PCI | percutaneous coronary intervention |
| CCS | chronic coronary syndrome |
| ACS | acute coronary syndrome |
| HIST | high- to moderate-intensity statin treatment |
| LIST | low-intensity statin treatment |
| EPC | circulating early-outgrowth endothelial progenitor cells |
| FACS | fluorescence-activated cell sorting |
| FITC | fluorescein isothiocyanate |
| EC-CFU | endothelial cell colony-forming units |
| EOC | early outgrowth endothelial cells |
| ECFC | endothelial colony-forming cells |
| KDR | kinase domain receptor |
| Ac-LDL | acetylated LDL |
| SMPC | smooth muscle progenitor cells |
| PDGF-BB | platelet derived growth factor BB |
| FCS | fetal calf serum |
| PBMC | peripheral blood mononuclear cells |
| Dil-Ac-LDL | 1,19–dioctadecyl–3,3,39,39–tetramethyl indocarbocyanine–labelled acetylated low-density lipoprotein |
| PDGFR | anti human platelet-derived growth factor receptor |
| SEM | standard error of the mean |
